# Injury of Thigh and Contusion of Back

**Published:** 1887-12

**Authors:** 


### INJURY OF THIGH AND CONTUSION OF BACK.

### CASE III.

J—— K——, aged thirty-four, laborer. The patient was brought to the hospital by patrol about 9:30 A. M., suffering with a crushing injury of his right leg as far up as the knee, a railroad engine having passed over it. A contusion of the back was also found. He was almost exsanguinated, the patrolmen not having correctly applied the rubber constrictor. His pulse was 160 and very weak. He was pale, very thirsty, and at times unconscious.

Digitalis, aromatic spirits of ammonia, and whisky were administered without avail. At 11:30 A. M., as he did not improve, infusion was deemed advisable. The median basilic vein was opened and a warm saline solution injected, consisting of sodium chloride, 6 parts; sodium bicarb., 1 part; aquæ dest., 1000, at a temperature of 100 deg. F. Eight ounces of this solution were used. Only slight improvement was noticeable in the pulse after the infusion, owing probably to the small amount of fluid injected. An Esmarch's bandage was applied to the crushed tissues, as far as to the healthy, and remained in place until the following day (1:30 P. M.), when the limb was amputed at the junction of the lower with the upper two-thirds of the thigh and dressed in the usual way. The patient did not do well for two weeks, the flaps sloughed, permitting the bone to protrude. He complained much of pain in the back. The temperature ranged during this time between 99.5 deg. and 102 deg. F. At the end of this time an abscess was discovered in the small of the back. It was opened and drained. The temperature immediately subsided, and did not rise again above 99 deg.; his appetite increased and he improved in every way.

December 7.—Re-amputation of bone, removing about an inch and ahalf.

December 22.—The stump is nearly healed; an ulcer, size of a half dollar, remains, and is studded with healthy granulations.

